# Leaf Physiological Responses of Three Psammophytes to Combined Effects of Warming and Precipitation Reduction in Horqin Sandy Land, Northeast China

**DOI:** 10.3389/fpls.2021.785653

**Published:** 2022-01-04

**Authors:** Wen-Da Huang, Yuan-Zheng He, Huai-Hai Wang, Yuan-Zhong Zhu

**Affiliations:** ^1^Naiman Desertification Research Station, Northwest Institute of Eco-Environment and Resources, Chinese Academy of Sciences, Lanzhou, China; ^2^Key Laboratory of Stress Physiology and Ecology in Cold and Arid Regions, Northwest Institute of Eco-Environment and Resources, Chinese Academy of Sciences, Lanzhou, China; ^3^School of Life Sciences, University of Chinese Academy of Sciences, Beijing, China

**Keywords:** psammophytes, Horqin sandy land, warming, precipitation reduction, physiological

## Abstract

The decreasing precipitation with global climate warming is the main climatic condition in some sandy grassland ecosystems. The understanding of physiological responses of psammophytes in relation to warming and precipitation is a possible way to estimate the response of plant community stability to climate change. We selected *Lespedeza davurica*, *Artemisia scoparia*, and *Cleistogenes squarrosa* in sandy grassland to examine the effect of a combination of climate warming and decreasing precipitation on relative water content (RWC), chlorophyll, proline, and antioxidant enzyme activities. We found that all experimental treatments have influenced RWC, chlorophyll, proline, and antioxidant enzyme activities of three psammophytes. *L. davurica* has the highest leaf RWC among the three psammophytes. With the intensification of precipitation reduction, the decreasing amplitude of chlorophyll from three psammophytes was *L. davurica* > *C. squarrosa* > *A. scoparia*. At the natural temperature, the malondialdehyde (MDA) content of the three psammophytes under severe drought treatment was much higher than other treatments, and their increasing degree was as follows: *A. scoparia* > *C. squarrosa* > *L. davurica*. At the same precipitation gradient, the proline of three psammophytes under warming was higher than the natural temperature. The differences in superoxide dismutase (SOD) among the three psammophytes were *A. scoparia* > *L. davurica* > *C. squarrosa.* Moreover, at natural temperature, more than 40% of precipitation reduction was most significant. Regardless of warming or not, the catalase (CAT) activity of *A. scoparia* under reduced precipitation treatments was higher than natural temperature, while the response of *L. davurica* was opposite. Correlation analyses evidenced that warming (T) was significant in *L. davurica* and precipitation (W) was significant in *A. scoparia* and *C. squarrosa* according to the Monte-Carlo permutation test (*p* = 0.002, 0.004, and 0.004). The study is important in predicting how local plants will respond to future climate change and assessing the possible effects of climate change on sandy grassland ecosystems.

## Introduction

For nearly a hundred years, the global climate has been in a state of continuous warming. Between 1880 and 2012, the global average surface temperature rose by about 0.85°C, and the raise in China is higher than the global level, which is 0.91°C (Stocker et al., 2014). Climate warming has altered the precipitation pattern of many ecosystems (Dai, 2013; Trenberth et al., 2014; Zhao et al., 2019). With climate warming, the precipitation of sandy grassland ecosystem gradually decreases and water evaporation increases, especially during the growing season (Yao et al., 2014; Ma et al., 2018), which leads to the increase in land degradation area (Ma et al., 2018), making the sandy grassland ecosystem very fragile. The increase in temperature and the decrease in precipitation limit the growth and physiological characteristics of plants and affect the stability of plant community in sandy grassland (Luo et al., 2008; Xu et al., 2014; Chen et al., 2015).

In arid and semiarid areas, temperature, and precipitation are the main climatic factors controlling individual growth and development (Xu et al., 2014; Yue et al., 2016; Chen et al., 2017). In particular, climate changes, such as heat and drought, may alter key physiological and biochemical characteristics of plants, for example, water use efficiency, enzyme activity, and chlorophyll indexes (Anjum et al., 2011; Din et al., 2011; Xu et al., 2014; Guo et al., 2016; Yun et al., 2016). In the long-term evolution process, desert plants have constantly improved their ability to respond to harsh environments (Xu and Zhou, 2006). Desert plants with different life forms have different adaptation strategies, especially adaptation of shrubs and herbs to multiple stressors is even more remarkable (Volder et al., 2010). At the same time, global or regional climate change will transform the dynamics, composition, and diversity of plant communities. Climate warming may significantly influence plant carbon assimilation, which could alter community composition (Peng et al., 2017). The seasonal and spatial changes in precipitation patterns determine the niche differentiation of plant communities (Engelbrecht et al., 2007). From the point of view of ecology and evolution, the adjustment of adaptive strategies of dominant species in plant communities is very important to maintain vegetation stability, as they both provide effective buffers against rapid climate change and contribute to long-term adaption (Hu et al., 2020; Zuo et al., 2020).

Horqin sandy land, a representative sensitive ecological region, is located in the agropastoral transitional zone between the Inner Mongolian Plateau and the Northeast Plains (42°41′–45°45′ N and 118°35′–123°30′ E) and is one of the four largest sandy areas in northern China; it covers an area of approximately 139,300 km^2^, which had been desertified sandy land area up to 71,884 km^2^ (Wang, 2003; Zhao et al., 2003). Landscape in this area is characterized by sand dunes that alternate with gently undulating lowland areas (Li et al., 2005). This area belongs to the continental semiarid monsoon climate and is in the temperate zone, with a mean annual temperature (AMT) of 3–7°C and mean annual rainfall (AP) of 350–500 mm (Zhao et al., 2003). Meanwhile, the increase in potential evapotranspiration would be more obvious than precipitation with increasing temperature in the future, which leads to a more severe water deficit and exaggerated the aridification or desertification of this area (Su et al., 2018). These altered climatic conditions could be expected to increase the vulnerability of sandy grassland ecosystems and may cause strong effects on the biological processes of native plants in the coming decades. Therefore, the protection of local dominant sandy plants plays an important role in maintaining community stability, controlling desertification, and protecting biodiversity.

The study of responses to climate change (warming and precipitation reduction) may contribute to understanding physiological and ecological acclimations of psammophytes in sandy grassland. Until now, some preliminary studies on drought-induced effects on physiological adaptations of psammophytes have been reported in Horqin sandy grassland (Luo et al., 2011; Chen et al., 2019). However, our understanding of the combined effects of future scenarios of warming and precipitation reduction on physiological responses of psammophytes in Horqin sandy grassland is scarce. Thus, the objectives of this study were (1) to determine whether there are differences in the physiological responses of the three psammophytes from the same sandy grassland community to warming and precipitation reduction gradients and (2) to examine the adaptive mechanisms of the three species to a sand environment change. The study is important in predicting how local plants will respond to future climate change and assessing the possible effects of climate change on sandy grassland ecosystems.

## Materials and Methods

### Study Site

The study was conducted at the Naiman Desertification Research Station (42°58′ N and 120°43′ E), Chinese Academy of Sciences, which is located in the southeastern of Horqin sandy land, eastern Inner Mongolia, China. The study area belongs to the typical temperate semiarid continental monsoon climate. The average annual precipitation was 351.7 mm (Liu et al., 2011), with uneven spatial and temporal distribution, in which the precipitation from June to September accounted for about 80%. The annual average temperature is 5.8–6.4°C. The natural vegetation mainly consists of *Salsola collina*, *Tribulus terrestris*, *Artemisia halodendron*, *Cleistogenes squarrosa*, *Setaria viridis*, *Bassia dasyphylla*, *Chenopodium acuminatum*, *Stipa klemenzii*, *Caragana microphylla*, *Artemisia scoparia*, *Lespedeza davurica*, and so on.

### Experimental Design

**FIGURE 1:**
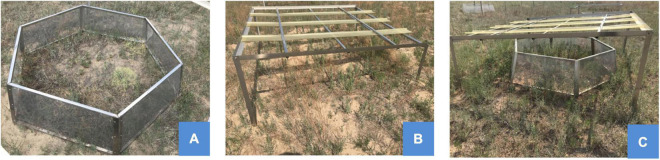
The experimental apparatus for this study. **(A)** Warming apparatus, **(B)** precipitation reduction apparatus, and **(C)** warming and precipitation reduction union apparatus.

We selected a typical sandy grassland where vegetation types and microtopography were fairly consistent and fenced with guardrail wire to prevent disturbance from wild animals and human activities. The experiment is a randomized complete block design with temperature and precipitation as treatment factors, which was established for simulating the most likely climate scenarios (warming and precipitation reduction) in the future. The device was 2.5 m × 2.5 m × 1.5 m. The structure had a square steel frame, with the top tilted by 15° to support rain intercept grooves for precipitation reduction (Figure 1). The rain intercept groove was made of highly transparent 5 mm-thick Perspex (Tongliao Xinlong Trading Co., Ltd.) with a light transmission. We followed the methods suggested by Marion et al. (1997) and used open-top chambers (OTCs) as a warming device. The OTCs were constructed of thick translucent glasses and six aluminum alloy columns. The material of glasses had high solar transmittance. The average atmospheric temperature increased by about 2.1°C during the growing season in the device. According to the annual precipitation data for the last 49 years in this area, the maximum precipitation reached 567.1 mm in 1986, which was 61.2% over the average, and the minimum precipitation was 213.1 mm in 2000, which was 39.4% less than the average precipitation. Temperature × precipitation treatments were set up as follows: (1) T_0_ (natural temperature) × W_0_ (natural precipitation), (2) T_0_ × W_20_ (precipitation decreased by 20%), (3) T_0_ × W_40_ (precipitation decreased by 40%), (4) T_0_ × W_60_ (precipitation decreased by 60%), (5) T (warming) × W_0_, (6) T × W_20_, (7) T × W_40_, and (8) T × W_60_. To avoid mutual interference, there were buffer strips of 2 m between the plots. Each treatment consisted of six replicate plots.

### Sampling

In July 2020, a vegetation survey was conducted on all sample plots. According to the survey data, we selected three dominant psammophytes with high species number, coverage, and biomass from shrub layer and herb layer, respectively, to determine the combined effects of simulated warming and precipitation reduction on their physiological responses. Among the three psammophytes we selected, *L. davurica* was the dominant species in the shrub layer, *A. scoparia* and *C. squarrosa* were the dominant species in the herb layer. The three psammophytes were widely distributed species in Horqin sandy land, and they were also important species in the composition of a sandy plant community.

Leaves of *A. scoparia*, *L. davurica*, and *C. squarrosa* were randomly cut on July 20, 2020. Several of them were taken immediately to the laboratory to measure the relative water content (RWC); the rest were placed in a liquid nitrogen tank for observation of enzyme activities and osmoregulatory substances. In addition to RWC, there were 6 physiological indexes tested using the Solarbio kit (Solarbio, Beijing, China), which were chlorophyll, malondialdehyde (MDA), proline, cellular superoxide dismutase (SOD), peroxidase (POD), and catalase (CAT).

### Data Analysis

SPSS 25.0 software (www.ibm.com/software/analytics/spss/) was used for statistical analysis. Before the ANOVA, the normality test was carried out to ensure that the experimental data conform to the normal distribution. We used ANOVA to test for significant differences among the treatments. A least significant difference (LSD) test was performed to determine any differences in warming and precipitation reduction treatments among the three species. Significant differences were defined at *p* < 0.05. Redundancy analysis (RDA) was used to determine the relative contribution of the measured environmental variables to physiological indices of the three psammophytes. Data were first analyzed using detrended correspondence analysis, which indicated that RDA was an appropriate approach (gradient length < 3). To avoid overfitting in the regression model due to the large number of explanatory variables, the most discriminating variables were selected using the “forward selection” procedure of the program during analysis. Physiological indices and environmental data were log (*x* + 1) transformed prior to analysis. RDA was performed using CANOCO Version 4.5 (www.downxia.com) (ter Braak and Smilauer, 2002). All graphs were drawn using Origin Software (2018) (www.downxia.com).

## Results

### Changes in Leaf Relative Water Content and Total Chlorophyll Content

**FIGURE 2:**
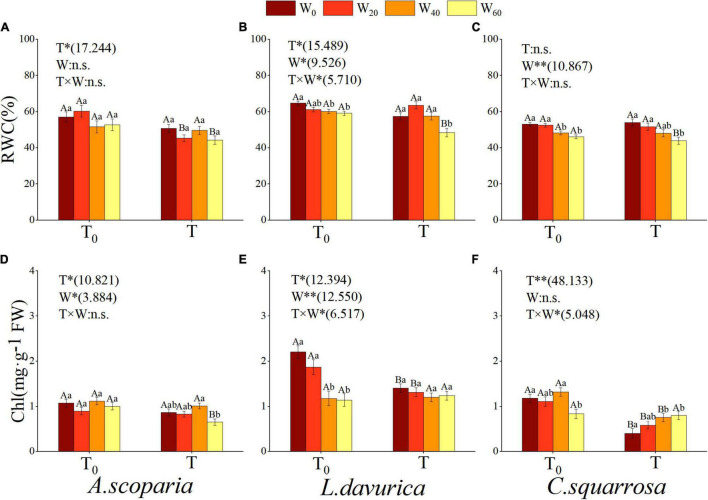
Influence of different simulating climate change treatments on the leaf relative water content (RWC) and chlorophyll content (Chl) in three psammophytes. Vertical bars denote the standard error. T_0_, natural temperature; T, warming; W_0_, natural precipitation, W_20_, W_40_, and W_60_, precipitation decreased by 20%, 40%, and 60%. * and ^**^, significant difference at *p* < 0.05 and *p* < 0.01 levels; n.s., no significant difference. Bars labeled with different lowercase letters represent significant differences among precipitation reduction treatments (*p* < 0.05). Bars labeled with different capital letters differ significantly between temperature treatments (*p* < 0.05). **(A–C)** indicate leaf relative water content of *A. scoparia*, *L. davurica*, and *C. squarrosa*, **(D–F)** indicate leaf chlorophyll content of *A. scoparia*, *L. davurica*, and *C. squarrosa*.

Warming, precipitation reduction, and hydrothermal interaction had influenced leaf RWC of the three psammophytes (Figures 2A–C). The results showed that warming had a significant effect on leaf RWC of *A. scoparia* and *L. davurica* (*p* = 0.003), precipitation reduction had a distinct impact on that of *L. davurica* and *C. squarrosa* (*p* ≤ 0.05), and hydrothermal interaction had significant effect only on *L. davurica* (*p* = 0.008) (Figures 2A–C). Warming significantly reduced the leaf RWC of the three plants, when precipitation was reduced by 60% (T × W_60_ vs. T_0_ × W_60_). At the same precipitation gradient, the RWC of *L. davurica* was higher than that of *C. squarrosa*, and the value of both plants decreased with the aggravation of drought stress, with a marked decrease of 8.54% and 13.11% (T_0_ × W_60_ vs. T_0_ × W_0_), respectively (Figures 2A–C).

Our results showed that warming remarkably impacted the total chlorophyll content of the three psammophytes (*A. scoparia*, *p* = 0.003; *L. davurica*, *p* = 0.002; *C. squarrosa*, *p* < 0.001) (Figures 2D–F), precipitation reduction notably affected that of *A. scoparia* (*p* < 0.05) and *L. davurica* (*p* < 0.001), and hydrothermal interaction remarkably influenced that of *L. davurica* and *C. squarrosa* (*p* < 0.05) (Figures 2D–F). As drought stress intensifies in natural temperature, total chlorophyll content of *L. davurica* showed a downward trend. The results of precipitation reduction less than 20% were significantly higher than those of precipitation reduction more than 40% (Figure 2E). As the synergistic effect of temperature and drought stress intensified, total chlorophyll content of *C. squarrosa* showed an upward trend, and the result in natural temperature was significantly lower than that of precipitation reduction of more than 40% (Figure 2F). At the same precipitation gradient, total chlorophyll content of the three psammophytes in natural temperature was higher than warming. The changes in total chlorophyll content in the three psammophytes were not consistent under different treatments. In warming, the content was decreased by 24.90% in *A. scoparia* and increased by 101.25% in *C. squarrosa* (T × W_60_ vs. T × W_0_). In natural temperature, the content was decreased by 48.01% in *L. davurica* and 29.25% in *C. squarrosa* (T_0_ × W_60_ vs. T_0_ × W_0_) (Figures 2D–F).

### Changes in Leaf Malondialdehyde and Free Proline Content

**FIGURE 3:**
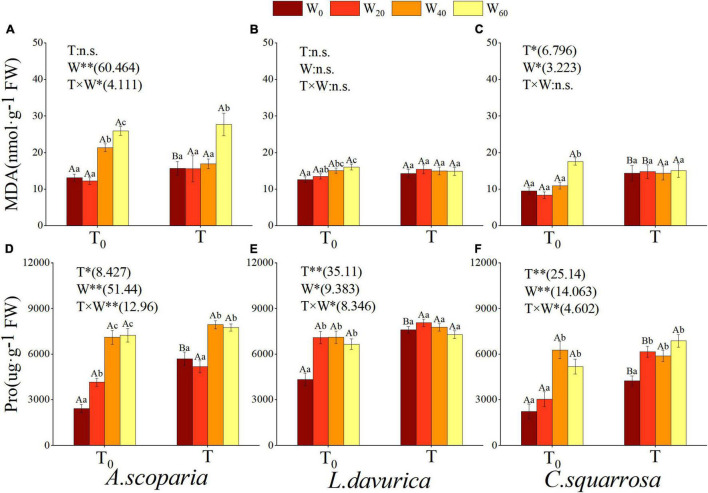
Influence of different simulating climate change treatments on the leaf malondialdehyde (MDA) and the proline content (Pro) in three psammophytes. Vertical bars denote the standard error. T_0_, natural temperature; T, warming; W_0_, natural precipitation, W_20_, W_40_ and W_60_, precipitation decreased by 20%, 40% and 60%. * and **, significant difference at *p* < 0.05 and *p* < 0.01 levels; n.s, no significant difference. Bars labelled with different lowercase letters represent significant differences among precipitation reduction treatments (*p* < 0.05). Bars labelled with different capital letters differ significantly between temperature treatments (*p* < 0.05). **(A–C)** indicate leaf malondialdehyde content of *A. scoparia*, *L. davurica*, and *C. squarrosa*, **(D–F)** indicate leaf proline content of *A. scoparia*, *L. davurica*, and *C. squarrosa*.

The MDA content of *A. scoparia* was significantly affected by precipitation reduction and hydrothermal interaction (*p* < 0.05) (Figure 3A). Warming and precipitation reduction had a notable impact on the MDA content of *C. squarrosa* (*p* < 0.05) but not on *L. davurica* (*p* > 0.05) (Figures 3B,C). With decreasing precipitation in natural temperature, the MDA content of the three psammophytes had similar changes. There was no difference in the three psammophytes between natural temperature and precipitation reduction by 20% (T/T_0_ × W_0_ vs. T/T_0_ × W_20_), and the maximum value was reached when precipitation was reduced by 60% (T_0_ × W_60_), which was significantly higher than other treatments, with an increase by 97.42% in *A. scoparia*, 28.12% in *L. davurica*, and 84.95% in *C. squarrosa* (Figures 3A–C). Only the MDA content of *A. scoparia* was affected by hydrothermal interaction, that is, the value reached the highest at T × W_60_ and was significantly higher than that of other treatments (Figure 3A).

The results showed that warming, precipitation reduction, and hydrothermal interaction all had a prominent effect on the proline content (Pro) of the three psammophytes (*p* < 0.05) (Figures 3D–F). Warming significantly increased the leaf Pro of the three psammophytes when the precipitation was natural (T × W_0_ vs. T_0_ × W_0_). At the same precipitation gradient, the Pro of the three psammophytes in warming was higher than the natural temperature. At natural temperature, precipitation reduction had a similar effect on the Pro in the three psammophytes, the Pro of precipitation reduction of more than 40% were significantly higher than those of natural precipitation, and the biggest difference reached 220.23, 64.08, and 88.99% in *A. scoparia*, *L. davurica*, and *C. squarrosa*, respectively. The results revealed that the effects of hydrothermal interaction in the protein content of *A. scoparia* and *C. squarrosa* are similar to the above one, the biggest difference in them reached 27.19 and 61.75%, respectively (Figures 3D,F).

### Changes in Leaf Antioxidant Enzymes Activity

**FIGURE 4:**
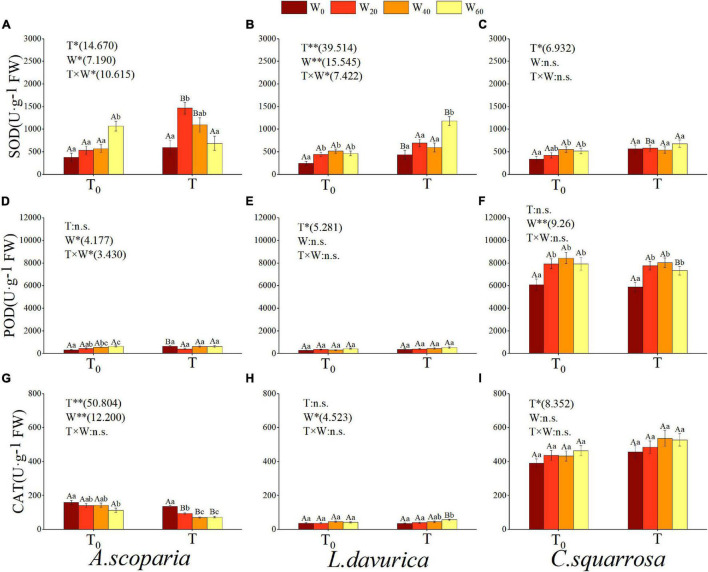
Influence of different simulating climate change treatments on leaf antioxidant enzymes in three psammophytes. Vertical bars denote the standard error. T_0_, natural temperature; T, warming; W_0_, natural precipitation, W_20_, W_40_ and W_60_, precipitation decreased by 20%, 40% and 60%. * and **, significant difference at *p* < 0.05 and *p* < 0.01 levels; n.s, no significant difference. Bars labelled with different lowercase letters represent significant differences among precipitation reduction treatments (*p* < 0.05). Bars labelled with different capital letters differ significantly between temperature treatments (*p* < 0.05). **(A–C)** indicate leaf superoxide dismutase content of *A. scoparia*, *L. davurica*, and *C. squarrosa*, **(D–F)** indicate leaf peroxidase content of *A. scoparia*, *L. davurica*, and *C. squarrosa*, **(G–I)** indicate leaf catalase content of *A. scoparia*, *L. davurica*, and *C. squarrosa*.

Warming, precipitation reduction, and hydrothermal interaction all had a noteworthy influence on the SOD activity of *A. scoparia* and *L. davurica* (*p* < 0.05) (Figures 4A,B). Only warming had an outstanding effect on that of *C. squarrosa* (*p* < 0.05) (Figure 4C). The results showed that, under natural temperature, the effect of more than 20% of precipitation reduction on the SOD of the three psammophytes was greater than that of natural precipitation, and the biggest difference reached 178.06% and 63.34% (T_0_ × W_60_ vs. T_0_ × W_0_) in *A. scoparia* and *C. squarrosa*, respectively, and 108.55% (T_0_ × W_40_ vs. T_0_ × W_0_) in *L. davurica*. However, the effect of hydrothermal interaction on them is inconsistent. The SOD activity was the largest in T × W_20_ on *A. scoparia* and T × W_60_ on *L. davurica*, which reached an increase of 145.56% and 170.61%, respectively(Figures 4A–C).

Warming and hydrothermal interaction only had a significant effect on the POD activity of *L. davurica* (*p* = 0.028) and *A. scoparia* (*p* = 0.029), respectively (Figures 4D,E). Precipitation reduction had a significant impact on that of *A. scoparia* (*p* = 0.013) and *C. squarrosa* (*p* < 0.001), respectively (Figures 4D,F). Regardless of warming or not, the POD value of more than 20% of precipitation reduction in *C. squarrosa* was higher than other treatments, and its POD activity in all precipitation gradients was much higher than other psammophytes (Figures 4D–F).

Warming had a prominent influence on the CAT of *A. scoparia* (*p* = 0.001) and *C. squarrosa* (*p* = 0.008), and it significantly decreased leaf CAT contents of two psammophytes when the precipitation reduction was higher than 20% (T × W_20/40/60_ vs. T_0_ × W_20/40/60_). Precipitation reduction only has a strong influence on *L. davurica* (*p* = 0.015) (Figures 4G–I). Regardless of warming or not, as the drought stress increases, the CAT content of *A. scoparia* showed a decreasing trend, with a biggest decrease of 28.81% (T_0_ × W_60_ vs. T_0_ × W_0_) and 48.00% (T × W_40_ vs. T × W_0_) (Figures 4G–I). Similar to POD, the CAT activity of *C. squarrosa* in all precipitation gradients was much higher than other psammophytes (Figures 4G–I).

### Correlation Between Leaf Physiological Characters and Climatic Change

**FIGURE 5:**
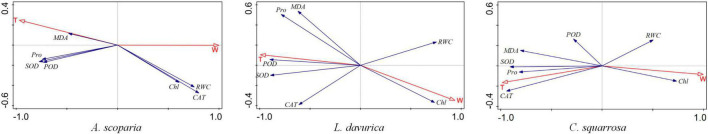
Correlation coefficients between leaf physiological characters and climatic change.

Physiological characters of the three psammophytes from different climatic scenarios in Horqin sandy land were influenced by warming (T) and precipitation reduction (W) (Figure 5). Correlation analyses evidenced that T and W togetherv explained 59.22% in *A. scoparia*, 78.53% in *L. davurica*, and 66.35% in *C. squarrosa* of the total variation in the data, with axes 1 and 2 explaining 53.24% and 5.98% in *A. scoparia*, 72.67% and 5.86% in *L. davurica*, and 64.85% and 1.5% in *C. squarrosa* of the total variation (Figure 5). Of the two environmental variables, T was significant in *L. davurica* and W was significant in *A. scoparia* and *C. squarrosa* according to the Monte-Carlo permutation test (*p* = 0.002, 0.004, and 0.004), whereas another variable was not significant according to the Monte-Carlo permutation test (*p* > 0.05). The total variation was explained by RDA, which was 59.22% in *A. scoparia*, 78.53% in *L. davurica* and 72.67% in *C. squarrosa*. This result indicates that some other climatic factors that were not considered in this study also contribute to the unexplained variation, and 53.2 and 64.4% was explained by W in *A. scoparia* and *C. squarrosa*, 71.9% was explained by T in *L. davurica* (Figure 5).

## Discussion

Temperature and precipitation play essential roles in plant growth and survival and deserve more attention in sandy grassland ecosystems. As psammophytes are currently subjected to a combination of stresses including warming and reduced precipitation, assessing their response to these stresses may be crucial to our understanding of stress tolerance mechanisms in psammophytes. In this study, we performed a primary study of responses of three psammophytes to the combination of warming and reduced precipitation. Our findings strongly suggested that three psammophytes have the capacity to physiologically adjust to warming and reduced precipitation conditions.

In general, environmental stress directly affects the physiological process of plant life by altering plant water status, affecting photosynthesis, destroying the balance of active oxygen metabolism in cells, and changing the capacity of osmotic adjustment (Lum et al., 2014). Specific indicators have leaf RWC, chlorophyll, MDA, and proline. In our study, these indexes of the three psammophytes have all undergone some changes with climate warming and precipitation reduction. Warming significantly reduced the RWC content of the three psammophytes at severe drought stress (precipitation reduction of 60%), indicating that their physiological adaptation was similar. Among the three psammophytes, *L. davurica* has the highest leaf RWC, indicating that it has the strongest water retention. At the same precipitation gradient, the chlorophyll values of the three psammophytes under natural temperature were higher than those under warming, which means that temperature increases limit their photosynthesis. In addition, the decrease in amplitude of the three psammophytes at natural temperature was *L. davurica* > *C. squarrosa* > *A. scoparia*. This reflects that photosynthetic capacity of different life forms differed in response to environment. At the natural temperature, the MDA content of the three psammophytes at severe drought treatment was much higher than other treatments, and their cell membrane damage degree was *A. scoparia* > *C. squarrosa* > *L. davurica*, indicating that shrubs are more adaptable to environmental changes than perennial herbs, which are more adaptable than biennial herbs. Different from the previous several indexes, at the same precipitation gradient, the Pro of three psammophytes under warming was higher than the natural temperature. These results declare that the hydrothermal interaction effects on the osmotic regulatory substances in the cytoplasm of the three dominant plants in the sandy grassland community are high. The Pro of these three plants was increased, indicating that the drought and heat resistance of psammophytes were improved. There are some studies that have been reported to support this concept (Zhou et al., 2014, 2015; Wang et al., 2016).

Antioxidant enzymes are important indicators of plant resistance to environmental stress, and the ability of plant resistance to stress can be evaluated according to its content. In our study, the antioxidant enzyme activities of the three psammophytes were different in response to temperature and precipitation change. There were differences in the change trends of the SOD activity among the three plants at natural temperature. The SOD activity of *L. davurica* increased significantly at mild drought stress, *C. squarrosa* increased significantly at moderate drought stress, and *A. scoparia* increased significantly at severe drought stress. This states that sandy grassland herbs inhibit membrane lipid peroxidation and maintain the metabolic balance of oxygen free radicals by increasing the SOD activity in cells, so as to adapt to drought, which is in accordance with the report of *Artemisia* plants (Wang et al., 2016). We found that the POD activity of *C. squarrosa* at all treatments was much higher than other two psammophytes. In addition, the POD activity was significantly higher at all precipitation reduction treatments than natural precipitation regardless of temperature. This result demonstrates that drought stress is the main environmental factor leading to the increase in *C. squarrosa* POD activity compared with temperature change, which is consistent with the research results of *S. viridis*, a suitable plant for sandy land (Chen et al., 2019). Similar to the results of POD, the CAT activities of *C. squarrosa* at all treatments were much higher than other two psammophytes. The CAT activity of *A. scoparia* at hydrothermal interaction treatments was lower than natural precipitation, while the response of *L. davurica* was the opposite. This phenomenon indicates that at the community level of sandy grassland, different life forms have different physiological regulation mechanisms to adapt to the high temperature and arid desert environment.

Correlation analyses evidenced that T and W together explained lower than 80% of the total variation in the data in the three psammophytes. This suggests that other biotic and abiotic factors that were not considered in this study may have contributed to the unexplained variation. Our study determined that W is the major factor that affects the physiological characters of *A. scoparia* and *C. squarrosa*, and T is the major factor that affects the physiological characters of *L. davurica*, suggesting that leaf physiological characters in herbs depend primarily on W and shrubs depend primarily on T. These results provide a scientific basis for species allocation in the process of vegetation restoration and reconstruction in sandy ecosystems. We must consider herbaceous plants as sand-fixing plants in areas with higher precipitation and soil moisture content, and shrubs as sand-fixing plants in areas with higher atmospheric and soil temperature. This helps to improve the stress tolerance of the species and vegetation stability.

## Conclusion

In this study, we found that the physiological responses of the three psammophytes to environmental gradients were different and related to plant life forms. The results showed that warming limits the photosynthesis of the three psammophytes. Biennial herbs have more photosynthetic capacity and cell membrane damage degree than perennial herbs and shrubs in sandy grassland communities. The hydrothermal interaction effects on the osmotic regulatory substances in the cytoplasm of the three psammophytes are great. Shrubs have better ability to maintain the metabolic balance of oxygen-free radicals than herbs under environmental stress. The data showed that W is the major factor that affects the physiological characters of herbs and T is the major factor that affects the physiological characters of shrubs. These results could provide a scientific basis for species allocation in the process of vegetation restoration and reconstruction in sandy ecosystems.

## Data Availability Statement

The raw data supporting the conclusions of this article will be made available by the authors, without undue reservation.

## Author Contributions

W-DH designed the experiment. W-DH and Y-ZH wrote the manuscript. Y-ZH performed the laboratory analysis. H-HW and Y-ZZ conducted the field trial. All authors contributed to the article and approved the submitted version.

## Conflict of Interest

The authors declare that the research was conducted in the absence of any commercial or financial relationships that could be construed as a potential conflict of interest.

## Publisher’s Note

All claims expressed in this article are solely those of the authors and do not necessarily represent those of their affiliated organizations, or those of the publisher, the editors and the reviewers. Any product that may be evaluated in this article, or claim that may be made by its manufacturer, is not guaranteed or endorsed by the publisher.
